# Systematic evaluation of patient-reported outcome (PRO) protocol content and reporting in UK cancer clinical trials: the EPiC study protocol

**DOI:** 10.1136/bmjopen-2016-012863

**Published:** 2016-09-21

**Authors:** Khaled Ahmed, Derek Kyte, Thomas Keeley, Fabio Efficace, Jo Armes, Julia M Brown, Lynn Calman, Chris Copland, Anna Gavin, Adam Glaser, Diana M Greenfield, Anne Lanceley, Rachel Taylor, Galina Velikova, Michael Brundage, Rebecca Mercieca-Bebber, Madeleine T King, Melanie Calvert

**Affiliations:** 1 Centre for Patient Reported Outcomes Research (CPROR), University of Birmingham, Birmingham, UK; 2 Institute of Applied Health Research, University of Birmingham, Birmingham, UK; 3 Health Outcomes Research Unit, Italian Group for Adult Hematologic Diseases (GIMEMA), Rome, Italy; 4 King's College London, London, UK; 5 UKCRC Registered CTU Network, University of Leeds, Leeds, UK; 6 Department of Heath Sciences, University of Southhampton, Southampton, UK; 7 NCRI Psychosocial Oncology and Survivorship CSG Consumer member, York, UK; 8 Queen's University Belfast, Centre for Public Health, Belfast, UK; 9 Leeds Institute of Cancer & Pathology, University of Leeds, Leeds, UK; 10 Sheffield Teaching Hospital NHS Foundation Trust, Sheffield, UK; 11 University College London, UCL EGA Institute for Women's Health, London, UK; 12 University College London Hospital (UCLH), London, UK; 13 University of Leeds, Leeds, UK; 14 Queen's Department of Oncology School of Medicine, Queen's Cancer Research Institute, Kingston, Ontario, Canada; 15 Faculties of Science and Medicine, University of Sydney, Sydney, New South Wales, Australia

**Keywords:** PROs, Quality of life, CONSORT PRO, SPIRIT Checklist, Evaluation, Cancer trials

## Abstract

**Introduction:**

Emerging evidence suggests that patient-reported outcome (PRO)-specific information may be omitted in trial protocols and that PRO results are poorly reported, limiting the use of PRO data to inform cancer care. This study aims to evaluate the standards of PRO-specific content in UK cancer trial protocols and their arising publications and to highlight examples of best-practice PRO protocol content and reporting where they occur. The objective of this study is to determine if these early findings are generalisable to UK cancer trials, and if so, how best we can bring about future improvements in clinical trials methodology to enhance the way PROs are assessed, managed and reported. Hypothesis: Trials in which the primary end point is based on a PRO will have more complete PRO protocol and publication components than trials in which PROs are secondary end points.

**Methods and analysis:**

Completed National Institute for Health Research (NIHR) Portfolio Cancer clinical trials (all cancer specialities/age-groups) will be included if they contain a primary/secondary PRO end point. The NIHR portfolio includes cancer trials, supported by a range of funders, adjudged as high-quality clinical research studies. The sample will be drawn from studies completed between 31 December 2000 and 1 March 2014 (n=1141) to allow sufficient time for completion of the final trial report and publication. Two reviewers will then review the protocols and arising publications of included trials to: (1) determine the completeness of their PRO-specific protocol content; (2) determine the proportion and completeness of PRO reporting in UK Cancer trials and (3) model factors associated with PRO protocol and reporting completeness and with PRO reporting proportion.

**Ethics and dissemination:**

The study was approved by the ethics committee at University of Birmingham (ERN_15-0311). Trial findings will be disseminated via presentations at local, national and international conferences, peer-reviewed journals and social media including the CPROR twitter account and UOB departmental website (http://www.birmingham.ac.uk/cpro0r).

**Trial registration number:**

PROSPERO CRD42016036533.

**Strengths and limitations of this study**

- Our review will assist the scientific community in determining how best to improve the way patient-reported outcomes (PROs) are assessed, managed and reported in future cancer trials.
- Our review will provide original data regarding the potential factors associated with PRO protocol quality and PRO reporting.
- The review will be limited to UK-led studies adopted to the National Institute for Health Research portfolio, which may limit generalisability of the results.
- Our selection criteria may lead to a cohort of studies that are not fully representative of the field as the trials may, on average, be of a higher quality than those found in the field (they are studies that have successfully completed) and they are more likely to be trials that have yielded a positive result (due to publication bias by journals).
- Our search criteria may be more likely to identify and obtain the required documents for non-industry sponsored trials, as the protocols of industry trials may be more likely to be confidential due to commercially sensitive information being included in them.

## Introduction

Patient-reported outcome (PRO) measures are validated questionnaires, self-completed by patients, that provide the patient perspective on physical, functional and psychological consequences of treatment and the degree and impact of disease symptoms.^1^ The value of using PROs in cancer clinical trials has been emphasised by major international health-policy and regulatory authorities and by patients with cancer.^2–4^ PRO results can inform patient choice and clinician decision-making, health technology assessment, health economic evaluations, labelling claims and healthcare policy and commissioning.^5–8^

Patients with cancer value PRO information and may use it to inform complex healthcare decisions.^9^ ^10^ For instance, PRO trial results can help patients to assess whether survival benefits of a new drug outweigh potential side effects or may assist patients and their clinicians in choosing between treatment options offering similar survival rates.^11–15^ Given their importance: (1) details regarding PRO assessment should be included in the trial protocol, to ensure appropriate data collection and management;^16^ ^17^ and (2) PRO results should be fully reported in arising trial publications, to enable timely access by patients, clinicians and policymakers and facilitate integration of findings into clinical practice.^18^ However, our recent review of 75 National Institute for Health Research (NIHR) Health Technology Assessment trials^19^ suggests important PRO information is frequently omitted from trial protocols, even where a PRO is the primary outcome of the study. This may lead to impaired data collection and result in poor quality PRO data, therefore limiting the potential of PRO findings to effectively inform patient care.^20^ Furthermore, international research suggests PRO results are poorly reported in trial publications or may not be reported at all.^18^ ^21^ ^22^ Thus, valuable information that may have a significant impact on treatment decision-making and outcomes may not be available to patients, clinicians and researchers. This would represent a waste of limited healthcare and research resources, which is unethical, and also devalues the contribution of trial participants who spend time and effort providing PRO data.^23^

This study aims to evaluate the standards of PRO-specific content in UK cancer trial protocols and their arising publications. The specific study objectives are to:

- Determine the completeness of PRO-specific protocol content in UK cancer trials.
- Determine the proportion of trials reporting PRO results, that is, the number of trials including PROs in their protocol and subsequently reporting the results in their principal publication.
- Determine the completeness of PRO reporting.
- Model factors associated with PRO protocol and reporting completeness, and with PRO reporting proportion.
- Highlight examples of best-practice PRO protocol content and reporting where they occur.

## Hypotheses

- Trials in which the primary end point is based on a PRO will have more complete PRO protocol and publication components than trials in which PROs are secondary end points.
- Publications of trials with more complete PRO protocol components will have more complete reporting of PROs than those of trials with less complete PRO protocol components.

## Methods and analysis

### Inclusion and exclusion criteria

**Figure 1:**
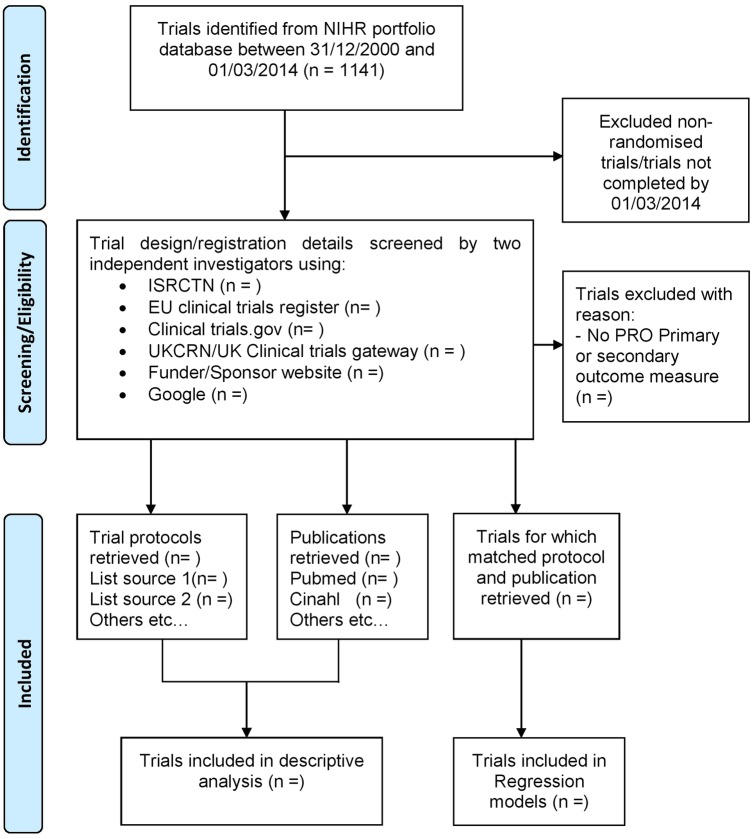
Schematic breakdown of trial search results of interventional clinical cancer trials between 1 March 2014 and 31 December 2000. PRO, patient-reported outcome.

Completed NIHR Portfolio Cancer clinical trials (all cancer specialities/age groups) will be included if they contain a primary/secondary PRO end point. The NIHR portfolio includes UK-led trials, supported by a range of funders, adjudged as high-quality clinical research studies.^24^ The sample will be drawn from studies completed between 31 December 2000 and 1 March 2014 (n=1141) to allow sufficient time for completion of the final trial report and publication (figure 1). Non-randomised trials and trials not completed by the cut-off date (1 March 2014) will be excluded.

### Protocol/publication sourcing

Two investigators (TK/KA) will independently search the NIHR Portfolio for eligible trials; disagreements will be resolved through discussion with a third or fourth reviewer (DK/MC). The most up-to-date trial protocol (final version approved by ethics) will be retrieved for review. Following a pilot, it was determined that trial registry information was frequently incomplete, therefore we plan to search for protocols using a range of resources outlined below:

1. NIHR portfolio; ISRCTN
2. EU Clinical Trials Register/EUdract
3. Clinical trials.gov
4. UKNCRN/UK Clinical trials gateway
5. Funder/Sponser
6. Google

For each protocol sourced, we will retrieve all related subsequent reports/publications either via a direct email to the named trial contact or using recognised bibliographic databases (eg, MEDLINE, Embase, CINAHL).

Publications will be sourced via direct contact with the corresponding/first author, or via the Patient-Reported Outcome Measures Over Time In Oncology (PROMOTION) Registry (http://promotion.gimema.it/),^25^ Embase; MEDLINE; Cinahl+; PsycINFO and Cochrane databases.

### Data extraction

Two investigators (TK/KA) will independently extract the following data for each included trial, using a predesigned data extraction form: trial demographics, year of protocol, the name(s)/type(s) of PRO(s) used, whether a PRO was a primary and/or secondary outcome, trial sample size, trial setting (national/international), number of research sites, funding source (including academic and industry supported), cancer specialty and age group (adult, teenage and young adult (TYA, 13–24 years inclusive), paediatric). The following data will be extracted from included trial reports/publications and recorded on the same form: year of final report/publication(s), number of publications and journal/publication source(s).

### Checklists

Completeness of the PRO-specific content of trial protocols will be assessed using a PRO protocol checklist^19^ detailing 33 PRO-specific items that are recommended in the literature for inclusion in a trial protocol. Completeness of general sections within each protocol will be assessed using the SPIRIT checklist, as a proxy measure of the overall strength of the protocol.^17^ ^26^ Completeness of PRO reporting will be evaluated using the CONSORT PRO extension checklist.^27^ We will also explore the use of the ISOQOL reporting standards checklist. General quality of reporting will be assessed by the 37-item 2010 CONSORT checklist.^28^

### Protocol and publication review

Trial protocols and their primary publications (presenting the principal study findings) will be evaluated in the review/analysis. Subsequent trial publications focused on PRO-specific results will also described. Two investigators (TK/KA) will independently assess the content of the included protocols using the SPIRIT and PRO protocol checklists. The investigators will independently assess consistency of the trial protocol/registration information with all subsequent publications to identify any discrepancies in the reporting of PRO trial outcomes. Unreported outcomes will be defined as those prespecified in the trial protocol/registration information but not reported in any subsequent publications.^29^ If a report is available, investigators will assess its completeness using the CONSORT and CONSORT-PRO Extension checklists (and the ISOQOL reporting standards checklist if applicable/feasible).^30^ Levels of investigator agreement will be determined for each checklist. Disagreements will be resolved through discussion with a third investigator (DK/MC).

### Quantitative data analysis

Descriptive analyses will be conducted on the number of SPIRIT and PRO checklist items present in the included trial protocols; on the proportion of incomplete PRO reporting and, where appropriate, on the number of CONSORT and CONSORT-PRO Extension checklist items present in the included in the principal trial publication. CONSORT-PRO checklist scores will also be described for those trials with subsequent PRO-specific publications and time from principal publication to secondary PRO publication will be reported.

#### PRO protocol and reporting models

To explore factors associated with the inclusion of PRO-specific protocol items, we will perform a prespecified multiple regression analysis in which the dependent variable will be the ‘PRO protocol checklist score’ and the independent variables will be (1) year of protocol, (2) whether the PRO was named as a primary or secondary outcome, (3) cancer specialty, (4) participant age group (adult/TYA/paediatric), (5) trial sample size, (6) funding source and (7) the SPIRIT checklist score. We will also perform a logistic regression, using the same covariates, in which the dependent variable will be ‘PRO trial results reported in the principal trial publication (yes/no)’.

To explore factors associated with the inclusion of CONSORT-PRO Extension reporting items, we will perform a prespecified multiple regression analysis in which the dependent variable will be the ‘CONSORT-PRO checklist score’ and the independent variables will be (1) the year of publication, (2) whether the PRO was named as a primary or secondary outcome, (3) whether there were single or multiple reports, (4) trial sample size, (5) funding source, (6) journal/publication source, (7) the standard CONSORT checklist score, and (8) the PRO protocol checklist score.

Where the sample size allows, we will attempt to conduct parallel exploratory analysis in which trials with a PRO primary end point are analysed separately from those with secondary PRO end points. All models and covariates will be finalised prior to the data analysis phase.

### Sample size calculation

A minimum of 80 protocols and publications will be required to satisfy the sample size requirement for the regression analyses (10 per covariate)^31^ of trials regardless of whether PROs are primary or secondary end points.

### Ethics and dissemination

The study was approved by the ethics committee at the University of Birmingham (ERN_15-0311) in September 2015. The results of this study will be disseminated via presentations at local, national and international conferences, peer-reviewed journals and through social media including the Centre for Patient Reported Outcomes Research twitter account and the University of Birmingham departmental website (http://www.birmingham.ac.uk/cpror), and via the NCRI (including the consumer forum), Macmillan Cancer Support and via international cancer trials groups.

### Protocol and registration

The study protocol is registered on PROSPERO (CRD42016036533), and registration details are available at: http://www.crd.york.ac.uk/PROSPERO/display_record.asp?ID=CRD42016036533.

## Discussion

A 2015 review investigated the frequency with which PROs (health-related quality of life) were specified in cancer trial protocols (2000–2003, non-UK) and subsequently reported in the literature.^21^ Of the 173 included trials, just over half (n=90) included a PRO, but only 35 of these (38%) reported the findings in an arising publication. However, no evaluation of the quality of PRO protocol/publication content was undertaken, and so the relationship between protocol completeness and reporting could not be assessed.

The current review is therefore necessary to provide a comprehensive representation of the standards of PRO content of protocols in UK cancer clinical trials and to assess whether addressing PRO content in protocols leads to more complete reporting of PROs in subsequent trial publications. Moreover, this systematic review will provide original data regarding the potential factors associated with PRO protocol quality and PRO reporting quality/proportion. This information will assist the scientific community in determining how best we can bring about methodological improvements in the way PROs are assessed, managed and reported in future cancer trials.
